# Hepatobiliary-Specific Contrast Agent in Biliary Leak

**DOI:** 10.5334/jbsr.1575

**Published:** 2018-09-10

**Authors:** Laurent Médart, Caroline Coibion, Jacques Deflandre

**Affiliations:** 1 Department of Radiology, CHR de la Citadelle, Liège, BE; 2 Department of Radiology, CHC, Liège, BE; 3 Department of Gastro-Enterology, CHR de la Citadelle, Liège, BE

**Keywords:** biliary leak, hepatobiliary-specific contrast

## Case Report

**Figure 1A and 1B:**
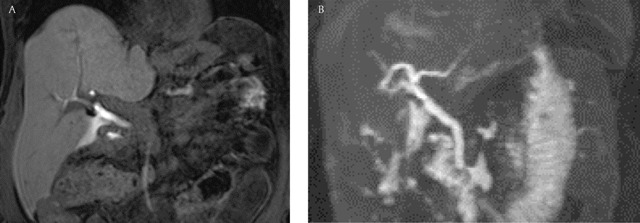


**Figure 2:**
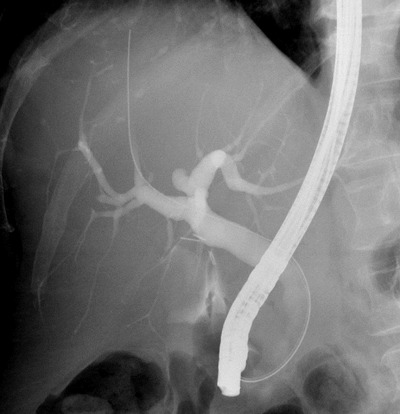


A 57-year-old woman was treated by cholecystectomy for gallbladder lithiasis. Coelioscopic surgery was uneventful and the patient was discharged the next day, but she came back to the hospital three days after surgery, with bilious fluid extruding from the right surgical orifice. Abdominal computed tomography (CT) showed a right subcutaneous fluid collection with a small biloma at the site of cholecystectomy (not shown). For direct identification of the biliary breach, Magnetic Resonance Imaging (MRI) with hepatobiliary-specific contrast agent (Gadoxetic acid – Primovist®) was performed. Only the hepatobiliary phase (i.e. one hour after intravenous injection of the MRI contrast at bedside) was performed using tridimensional fat-saturated T1-weighted imaging. Opacification of the biliary ducts was excellent and opacified bile was depicted in the peritoneum. Thick reformations (based on 1.5 mm thickness FS T1 acquisition) located the biliary leak at the proximal portion of the common biliary duct (Figure 1A and 1B). The MRI findings were confirmed on endoscopic retrograde cholangiography, and the duct breach was treated by sphincterotomy and temporary covered stent placement (Figure 2).

## Discussion

Biliary leak is a rare post-surgical or post-traumatic event. The presence of a collection can be identified by cross-sectional imaging. Bile content versus fluid collection of other nature is less easy to predicate. Identification of the exact location of the biliary breach is of great importance and often requires invasive retrograde cholangiography. MRI is the most reliable non-invasive technique for biliary imaging. Although conventional T2-weighted cholangiography provides anatomic information, the exact site of a bile breach is not usually identifiable. Delayed contrast-enhanced MRI with hepatobiliary-specific contrast agent adds the ability to evaluate the site of bile leak. Gadoxetic acid (Gd-EOB-DTPA), Primovist®, is a hepatobiliary-specific contrast agent taken up by functioning hepatocytes with nearly 70% biliary excretion. Because of its paramagnetic properties, this agent causes T1-shortening of the liver and biliary tree on delayed T1-weighted images. In addition, fluid collections, ascites, and fluid-containing structures that can obscure biliary tree depiction on conventional T2-weighted MRCP are inherently erased on T1-weighted sequences [1].

MRI with hepatobiliary-specific contrast agent is a promising substitute to invasive cholangiography when a biliary leak is suspected. It allows definite demonstration of ongoing biliary leak, precise breach identification and ideal treatment planning.
